# Board of Education and the Tuberculous Child

**Published:** 1914-01-03

**Authors:** 


### BOARD OF EDUCATION AND THE TUBERCULOUS CHILD.

If there be one Blue Book which has a really definite claim to consideration by the thinking public it is surely the annual report by the chief medical officer of the Board of Education, Sir George Newman. After six years there are now more than a thousand doctors engaged in that special form of medical practice included under the generic term of "school work."

Of the general trend of the school hygiene movement it may safely be said that this is all towards betterment. From time to time in these columns we have called attention to the development of the sphere of the school medical officer. The workers in this branch of the public health service have not been without their grievances, and only recently they have taken steps to secure a more active co-operation with a view to improving their status. It is therefore of interest to see that Sir George Newman encourages the enlargement of the sphere of the school medical officer, and gives hints to local education authorities that it is more economical to offer adequate remuneration.

One of the outstanding features of the progress made in practical school hygiene is the fuller differentiation of abnormal children and the increasing tendency on the part of local authorities to modify the school curriculum in their behalf. There is much to be learned from handling the exceptional child as to the best way of dealing with normal children. Much time and labour are now being devoted to the mentally defective child, the frail or retarded child, and the tuberculous child. The subjects of the incidence and the treatment of tuberculosis in school children are naturally matters to which considerable attention is being paid at the present time. Now that the notification of tuberculosis of all forms is compulsory, it behoves the authorities to see that effective co-operation exists between the medical officer of health, the tuberculosis officer, and the school medical officer. Through these three channels, which in some areas do not mean three separate officials, it should be possible to sweep all the cases of tuberculosis in any given area into the administrative net. The tuberculosis dispensary in relation to school children will no doubt play a very important part. In most areas where such a dispensary is working the school medical officer refers all tuberculous or suspected cases to the tuberculosis officer, and through his organisation the "contacts" are got at. In the following up of cases these two officials can be of great mutual help. Co-ordination of this kind not only serves to provide the necessary supervision, but should in time yield data which will help to solve the difficulty of diagnosis and correct our present knowledge of the course of the disease.

The question of the pre-tuberculous child is one to which more attention will have to be paid, and here we find that the Board of Education are prepared to consider the preventive treatment of such cases on broad lines. Open-air schools are likely to loom larger in future educational policies. At present the few that have been started, though tentative, are being continued in every case. Most people will agree that so long as a tuberculous case is not a danger to others it is far more satisfactory to allow the child to attend school than to leave it amid the usually unhygienic surroundings of its home. At the school it may benefit under the Education (Provision of Meals) Act by the provision of wholesome meals and extra milk, and these will do more good than cod-liver oil towards restoring the resistant power of a delicate child who appears likely to contract the disease. Indeed, school attendance will prove a much more useful substitute for an open-air school than the comparatively uncontrolled freedom of home life.

